# Supplementary material for: Structural basis of S-adenosylmethionine-dependent allosteric transition from active to inactive states in methylenetetrahydrofolate reductase
Source: Nat Commun. 2024 Jun 17;15:5167. doi: 10.1038/s41467-024-49327-5 (PMC11183114; doi:10.1038/s41467-024-49327-5)
Supplement: Supplementary file 1 — Supplementary Information [file 41467_2024_49327_MOESM1_ESM.pdf]

**Structural basis of *S*-adenosylmethionine-dependent Allosteric Transition from Active to Inactive States in Methylenetetrahydrofolate Reductase**

**Kazuhiro Yamada[1,2‡*], Johnny Mendoza[1], Markos Koutmos[1,2*]**

[1] Department of Chemistry, University of Michigan, Ann Arbor, MI, 48109

[2] Program in Biophysics, University of Michigan, Ann Arbor, MI, 48109

‡Current Address: Department of Biological Chemistry, University of Michigan, Ann Arbor, MI, 48109

*To whom correspondence should be addressed: yamadak@umich.edu (co-corresponding), mkoutmos@umich.edu (corresponding)

**Supplementary Information**

Supplementary Figures

1 and 2. Identification of phosphorylation sites of recombinant wild-type *h*MTHFR

3 and 4. *h*MTHFR Arg357Cys patient mutation

5. FAD binding pocket in *c*MTHFR, R-state

6. Limited proteolysis of *c*MTHFR in the presence or absence of AdoMet

7. Limited proteolysis of *c*MTHFR$^{R315A}$ in the presence or absence of AdoMet

8. FAD and Tyr361 interaction in *c*MTHFR, T-state

9. AdoMet Binding sites in *c*MTHFR, T-state

10. Electron Density around AdoMet allosteric inhibitors in *c*MTHFR, T-State

11. Structural rigidity of *c*MTHFR domains in R- and T-states

12. R-State structure comparison: *c*MTHFR and *h*MTHFR

13. MTHFR Multiple Sequence Alignment

Supplementary Tables

Supplementary Table 1. X-Ray Data Collection and Refinement Statistics

Supplementary Table 2. Bacterial and insect strains, plasmids, and synthetic oligonucleotides used in this study

Supplementary Discussion

1. Identification of phosphorylation sites of recombinant wild-type *h*MTHFR
2. *h*MTHFR Arg357Cys patient mutation & Other Patient Mutations
3. Limited proteolysis of *c*MTHFR$^{wt}$ and *c*MTHFR$^{R315A}$ in the presence or absence of AdoMet
4. Structural rigidity of *c*MTHFR domains in R and T states

Supplementary References

## Supplementary Figures

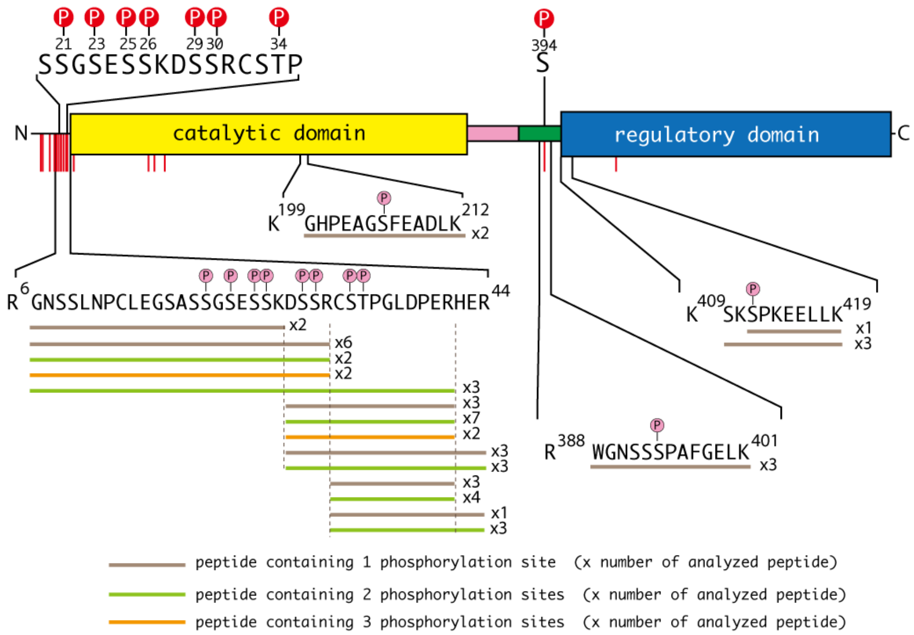


**Supplementary Figure 1. Schematic representation of the phosphorylation sites in recombinant *h*MTHFR.** The domain structure of *h*MTHFR is shown in a systematic mode. The catalytic and regulatory domains are shown in yellow and blue, respectively. The linker connecting the catalytic and regulatory domains is colored pink and green, denoting the "retractable-hinge region" and "velcro-wedge", respectively (see the main text for a detailed explanation). The upper part of the schematic domain illustration shows eight phosphorylated residues that are consistent in both the previous report and the present study. These residues are marked with red circled "P" letters. The previously reported phosphorylation sites are indicated by red vertical lines[2]. The phosphorylation sites identified in this study are described in the lower part. Tryptic fragments of *h*MTHFR were analyzed by LC-MS and phosphorylated amino acid residues identified in this study are marked with pink circled "P" symbols. The horizontal lines below the amino acid sequences represent the numerical distribution of the MS/MS fragments analyzed. Each color within these lines indicates the different amounts of phosphorylated amino acid residue(s) in a tryptic peptide, a classification explained in the lower section.

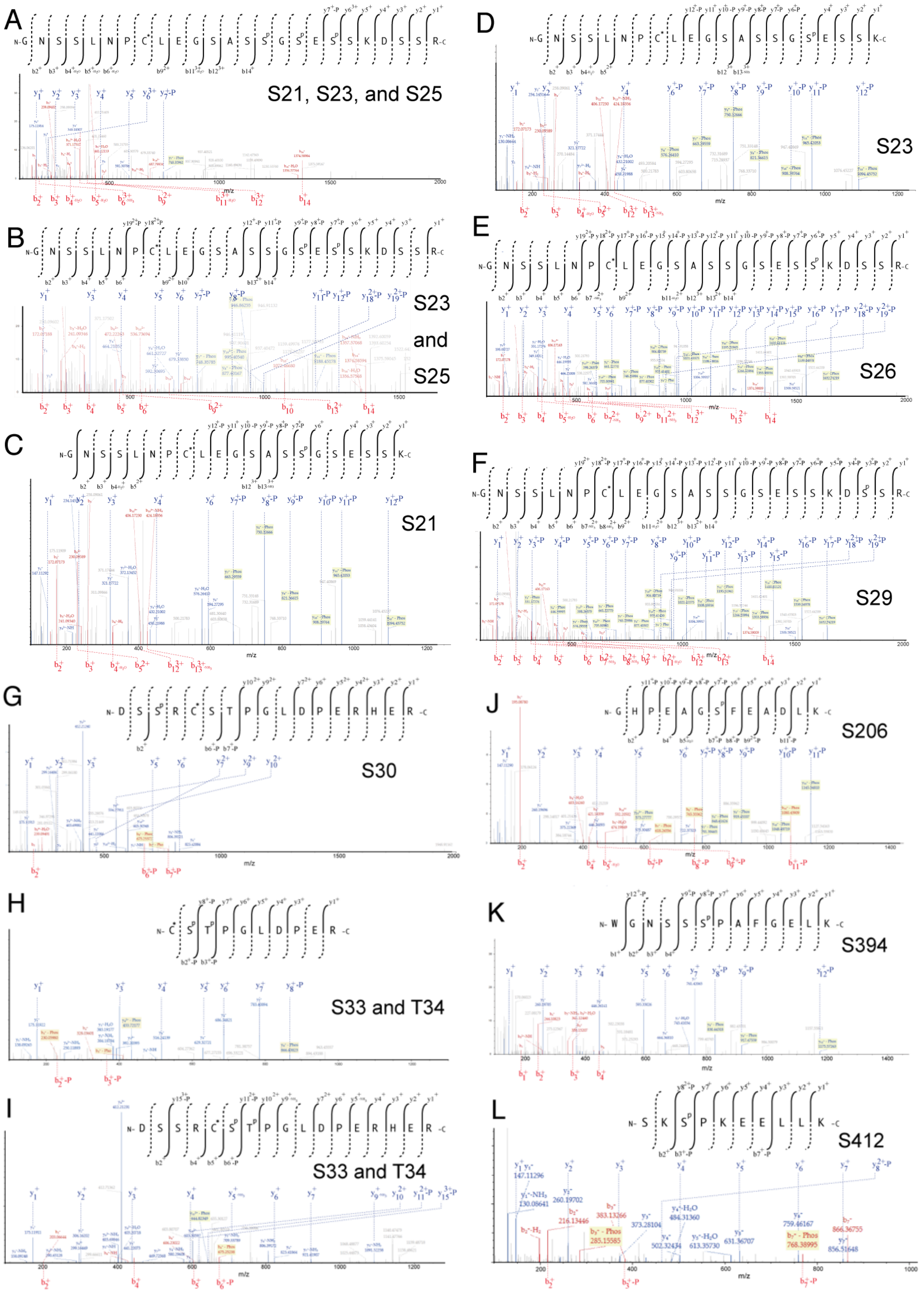


**Supplementary Figure 2. Identification of phosphorylation sites of recombinant *h*MTHFR by mass spectrometry.** (**a-l**) LC-MS/MS data showing Higher-energy collisional dissociation (HCD) -induced fragmentation mass spectra identifying eleven phosphopeptides. Observed b-ions are shown in red, whereas y-ions are shown in blue. **a** MS/MS spectrum of precursor m/z 914.00164 Da (+3) and MH + 2739.99038 Da, of the tryptic phosphopeptide GNSSLNP(*C)LEGSAS(pS)G(pS)E(pS)SKDSSR; where (*C) indicates carbamidomethylated Cys and (pS) represents for phosphorylated Ser. **b** MS/MS spectrum of precursor m/z 887.34474 (+3) and MH + 2660.01966 Da,

of the tryptic phosphopeptide GNSSLNP(*C)LEGSASSG(pS)E(pS)SKDSSR. **c** MS/MS spectrum of precursor m/z 712.29251 (+3) and MH + 2134.86297 Da, of phosphopeptide GNSSLNP(*C)LEGSAS(pS)GSESSK. **d** MS/MS spectrum of precursor m/z 712.29251 (+3) and MH + 2134.86297 Da, of the tryptic phosphopeptide GNSSLNP(*C)LEGSASSG(pS)ESSK. **e** MS/MS spectrum of precursor m/z 860.68966 (+3) and MH + 2580.05444 Da, of the tryptic phosphopeptide GNSSLNP(*C)LEGSASSGSES(pS)KDSSR. **f** MS/MS spectrum of precursor m/z 860.68966 (+3) and MH + 2580.05444 Da, of the tryptic phosphopeptide GNSSLNP(*C)LEGSASSGSESSKD(pS)SR. **g** MS/MS spectrum of precursor m/z 552.89477 (+3) and MH + 1656.66977 Da, of the tryptic phosphopeptide DS(pS)R(*C)STPGLDPER. **h** MS/MS spectrum of precursor m/z 646.22458 (+3) and MH + 1291.44189 Da, of the tryptic phosphopeptide (*C)(pS)(pT)PGLDPER. **i** MS/MS spectrum of precursor m/z 540.46488 (+3) and MH + 2158.83768 Da, of the tryptic phosphopeptide DSSR(*C)(pS)(pT)PGLDPERHER. **j** MS/MS spectrum of precursor m/z 719.30610 (+2) and MH + 1437.60492 Da, of the tryptic phosphopeptide GHPEAG(pS)FEADLK. **k** MS/MS spectrum of precursor m/z 730.31791 (+2) and MH + 1459.62854 Da, of tryptic phosphopeptide WGNSS(pS)PAFGELK. **l** MS/MS spectrum of precursor m/z 413.55088 (+3) and MH + 1238.63807 Da, of tryptic phosphopeptide SK(pS)PKEELLK.

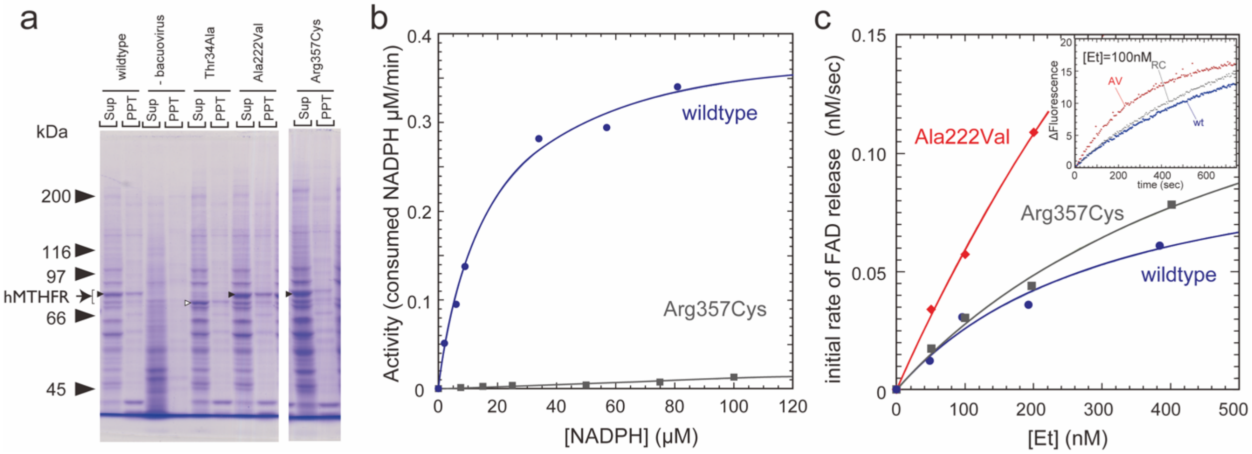


**Supplementary Figure 3. Heterologous expression, NADPH-menadione oxidoreductase activity, and FAD release of the Arg357Cys mutation. a** Insect cells, infected with baculovirus harboring *h*MTHFR genes, were lysed using a lysis buffer (10 mM Tris-HCl at pH 7.2, 2 mM ethylenediaminetetraacetic acid, 0.1 M NaCl, 50 mM sodium fluoride, 1 mM dithiothreitol, 1 mM phenylmethylsulfonyl fluoride, and 1% NP-40) on ice. The resulting cell lysate was subjected to centrifugation at 4˚C to obtain soluble (supernatant, Sup) and insoluble (precipitate, PPT) fractions, both of which were subsequently analyzed by SDS-PAGE. The gel was stained with Coomassie brilliant blue, with arrowheads indicating *h*MTHFR cDNA products. The Thr34Ala mutant (indicated by a white arrowhead) exhibited faster migration, due to its lack of post-translational modifications. "-baculovirus" indicates the extract from Sf9 cells not infected with baculovirus. **b** The NADPH-menadione oxidoreductase activity of wild-type (colored in blue) and the Arg357Cys mutant (colored in gray) *h*MTHFR was carried out at room temperature under varying concentrations of NADPH. Each reaction mixture contained 20 nM of *h*MTHFR. **c** FAD dissociation after dilution of wild-type and mutant *h*MTHFR. Released FAD was detected by fluorometry. The enzyme solution was incubated at 46 °C. The initial rate of FAD release is plotted against enzyme concentration after dilution for the wild-type and mutant enzymes: wild-type (blue), Ala222Val (red), and Arg357Cys (gray). The inset represents the change in fluorescence over time for wild-type and mutant enzymes diluted to 100 nM. Data are of representative experiments, which have been repeated ≥3 times.

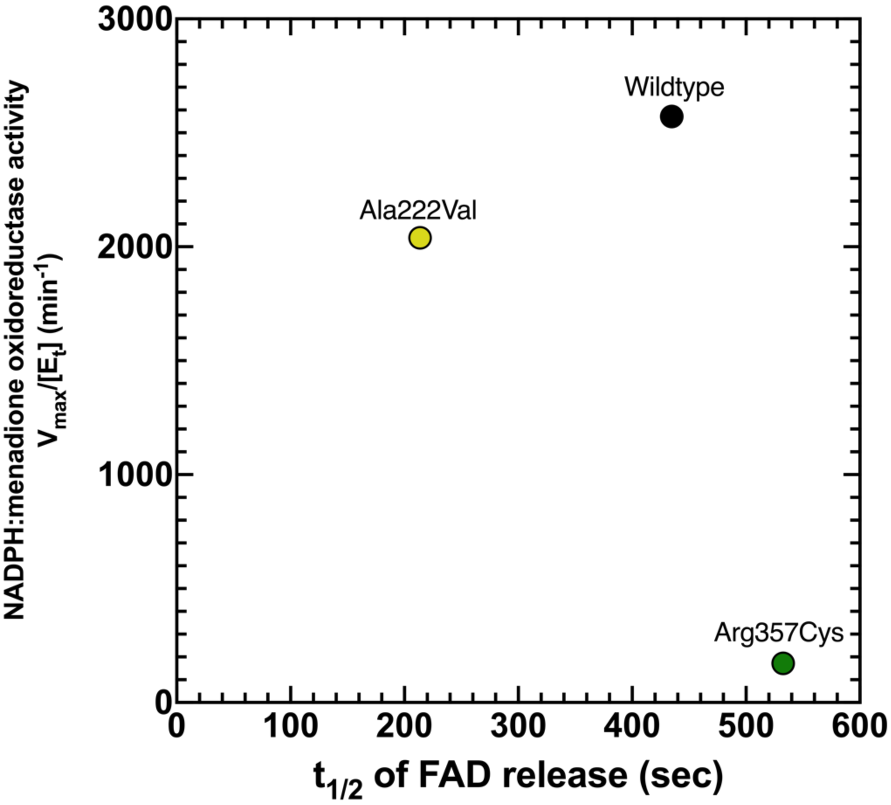


**Supplementary Figure 4. NADPH-menadione oxidoreductase activity versus FAD release of *h*MTHFR patient mutations.** The activities of *h*MTHFR wild-type (black) and patient mutants (catalytic domain, yellow; linker, green) versus half-life of FAD dissociation were plotted to ascertain trends that could allow for the rational capture of the elusive T-state. Note that the Arg357Cys mutant has greatly reduced activity and FAD retention that is similar to the wild-type. Data are from n=1 biological samples and were only used to ascertain any preliminary trends regarding FAD release and activity among patient mutants.

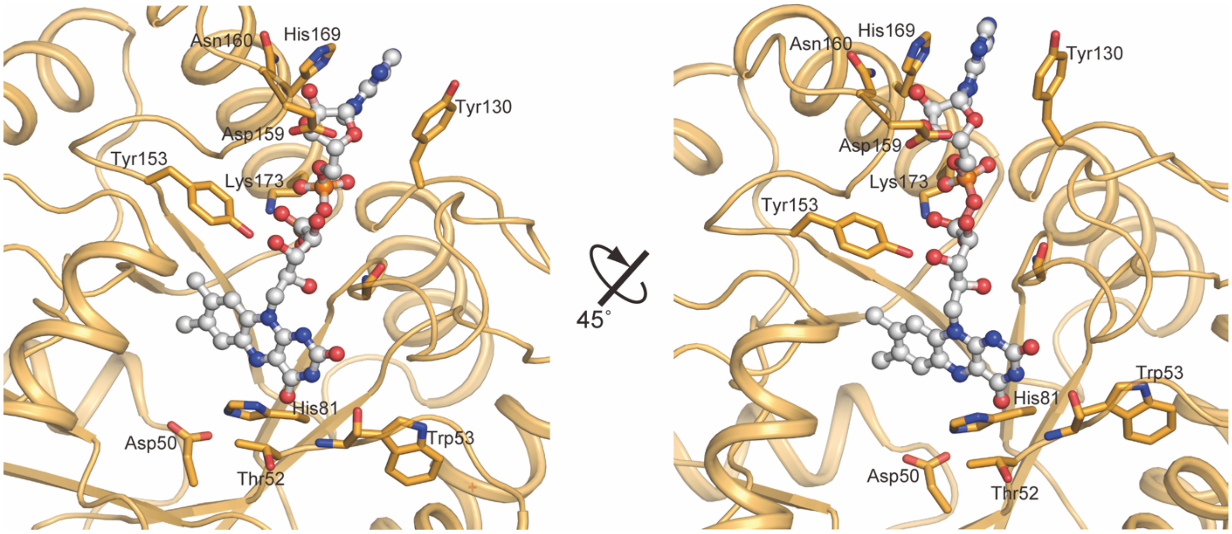


**Supplementary Figure 5. FAD binding site and mode in *c*MTHFR, R-State.** The FAD cofactor of *c*MTHFR is bound in an unoccluded state, making several interactions with the catalytic domain. Of note are the hydrogen bonding networks between N5 of FAD centered on conserved residues His81 and Asp50, along with a strong hydrogen bond between the universally conserved Thr52 and O4 of FAD. His169 and Tyr130 provide π-stacking interactions that serve to hold the adenine moiety in place.

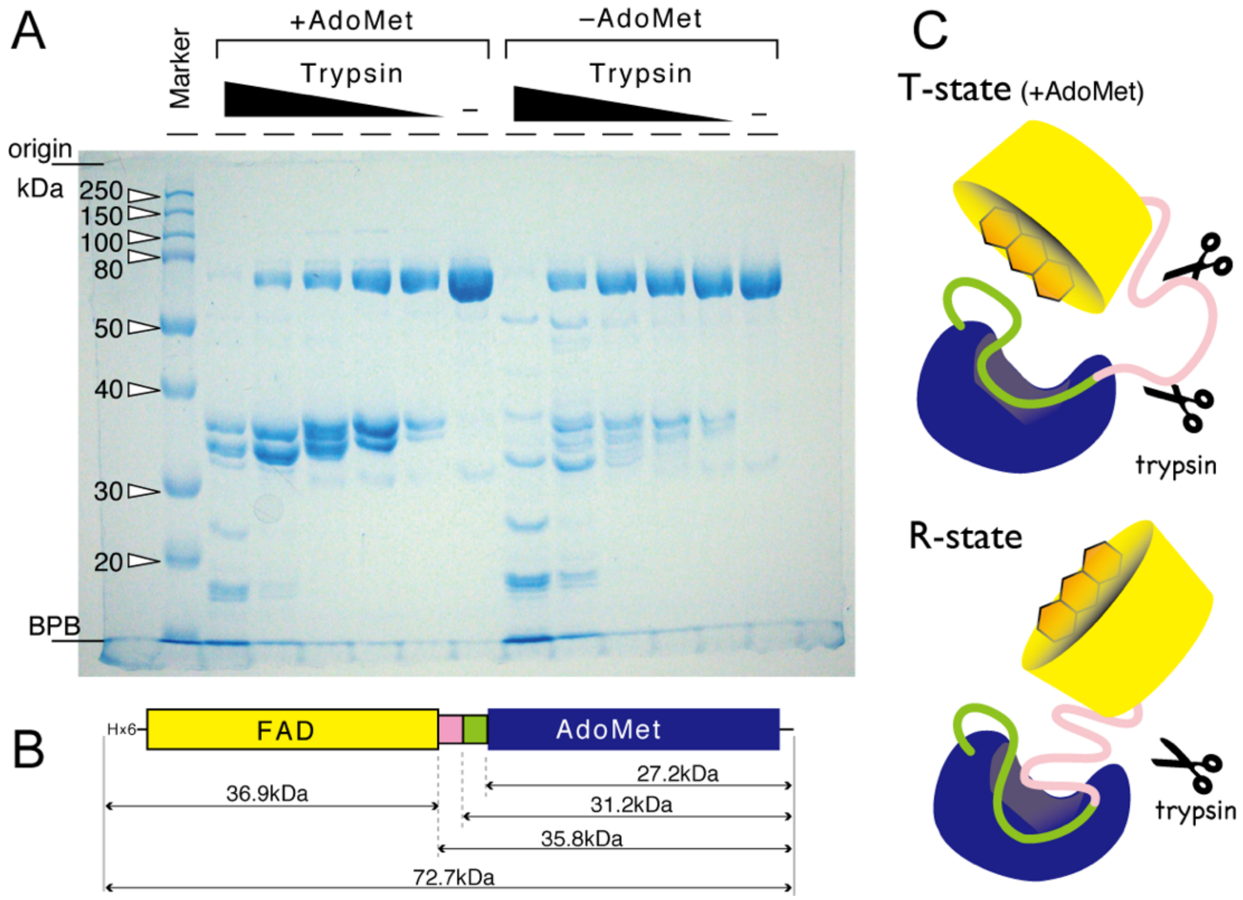


**Supplementary Figure 6. Limited Proteolysis of *c*MTHFR$^{wt}$.** **a** A quantity of 14 µg of purified *c*MTHFR$^{wt}$ was incubated with varying amounts of trypsin (from ~2 µg to ~0.2 ng) for 20 min at room temperature. The reaction was carried out both in the absence as well as in the presence of 100 µM AdoMet. Trypsin activity was quenched after the reaction by the addition of sample buffer for SDS-PAGE containing 1% SDS followed by heating at 95 ˚C for 10 minutes. The resulting *c*MTHFR$^{wt}$ fragments were separated by SDS-PAGE and visualized by Coomassie brilliant blue staining. **b** The schematic representation of the domain structure of *c*MTHFR is shown, along with the theoretical molecular weight of each domain. The catalytic domain (yellow) and the regulatory domain (blue) accommodate the FAD cofactor and AdoMet, respectively. Two colors, pink and green, in the linker region represent the "retractable region" and the "inverted cap-for-active-site", respectively. **c** The protein conformations of *c*MTHFR in the T- and R-states are shown in cartoon mode. The color scheme is the same as in panel B. In the R-state, the linker is intricately folded between the catalytic and regulatory domains. Conversely, in the T-state, the retractable region is exposed to the solvent, facilitating trypsin access to the solvent-exposed retractable region, and allowing cleavage of the linker region in the T-state (shown in **c**, top). In contrast, protease access to the linker is difficult when the retractable region is folded in the R-state (shown in **c**-bottom). Data are of representative experiments, which have been repeated ≥2 times. Cartoons in Panel **c** were created with BioRender.com released under a Creative Commons Attribution-NonCommercial-NoDerivs 4.0 International license.

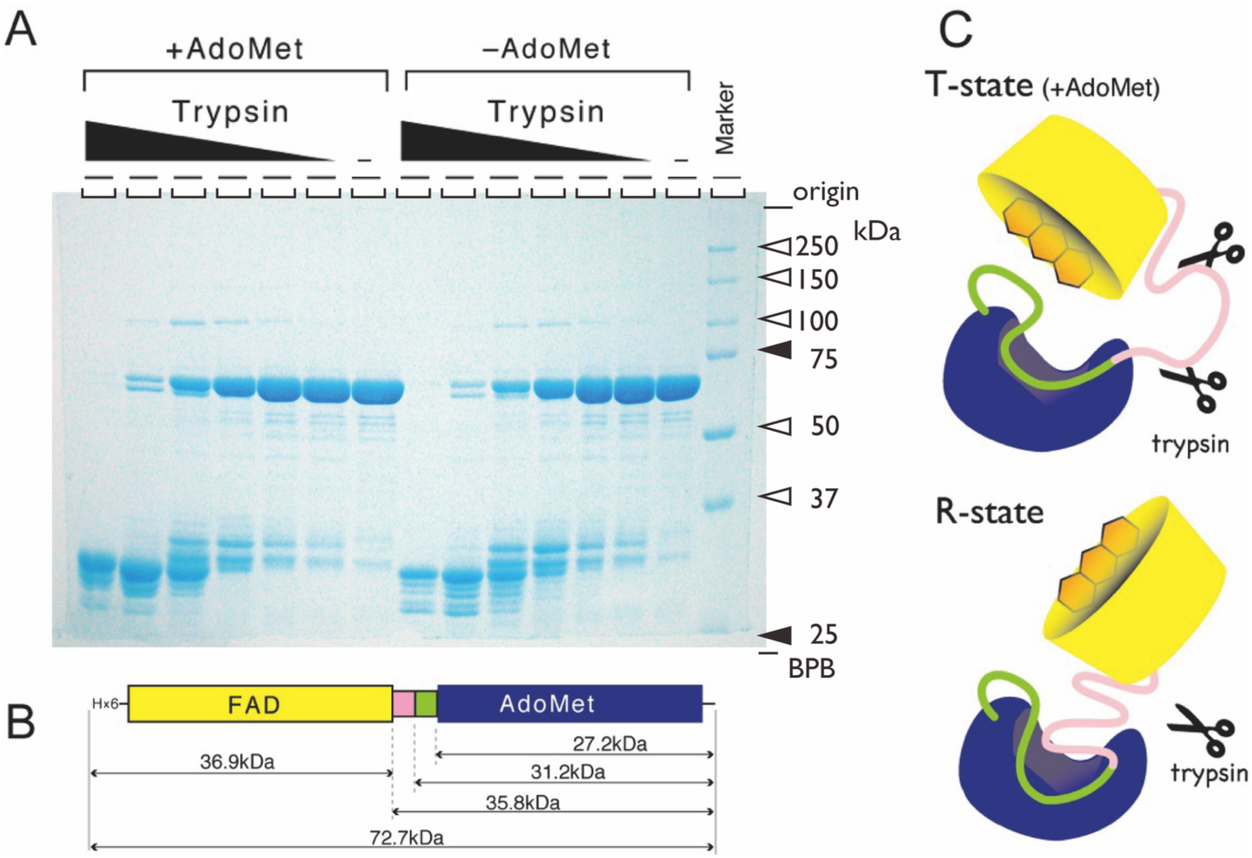


**Supplementary Figure 7. Limited Proteolysis of *c*MTHFR$^{R315A}$**. **a** A quantity of 14 µg of purified *c*MTHFR$^{R315A}$ was incubated with varying amounts of trypsin (from ~2 µg to ~0.2 ng) for 20 min at room temperature. The reaction was carried out both in the absence as well as in the presence of 100 µM AdoMet. Trypsin activity was quenched after the reaction by the addition of sample buffer for SDS-PAGE containing 1% SDS followed by heating at 95 ˚C for 10 minutes. The resulting *c*MTHFR$^{R315A}$ fragments were separated by SDS-PAGE and visualized by Coomassie brilliant blue staining. **b** The schematic representation of the domain structure of *c*MTHFR is shown, along with the theoretical molecular weight of each domain. The catalytic domain (yellow) and the regulatory domain (blue) accommodate the FAD cofactor and AdoMet, respectively. Two colors, pink and green, in the linker region represent the "retractable region" and the "inverted cap-for-active-site", respectively. **c** The protein conformations of *c*MTHFR in the T- and R-states are shown in cartoon mode. The color scheme is the same as in panel B. In the R-state, the linker is intricately folded between the catalytic and regulatory domains. Conversely, in the T-state, the retractable region is exposed to the solvent, facilitating trypsin access to the solvent-exposed retractable region, and allowing cleavage of the linker region in the T-state (shown in **c**, top). In contrast, protease access to the linker is difficult when the retractable region is folded in the R-state (shown in **c**-bottom). Data are of representative experiments, which have been repeated ≥2 times. Cartoons in Panel **c** were created with BioRender.com released under a Creative Commons Attribution-NonCommercial-NoDerivs 4.0 International license.

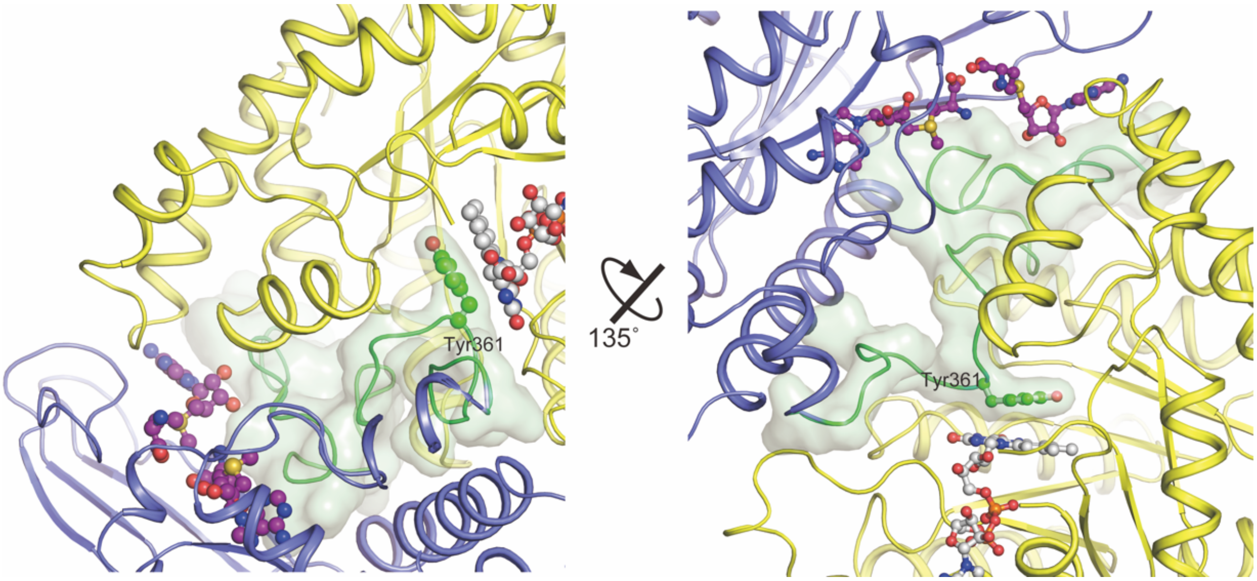


**Supplementary Figure 8. FAD binding site and mode in *c*MTHFR, T-State.** The FAD cofactor of *c*MTHFR is bound in an occluded state. Tyr361 of the velcro-wedge region of the linker provides a π-stacking interaction that serves to hold the FAD isoalloxazine moiety in place and occludes the *si*-face of FAD.

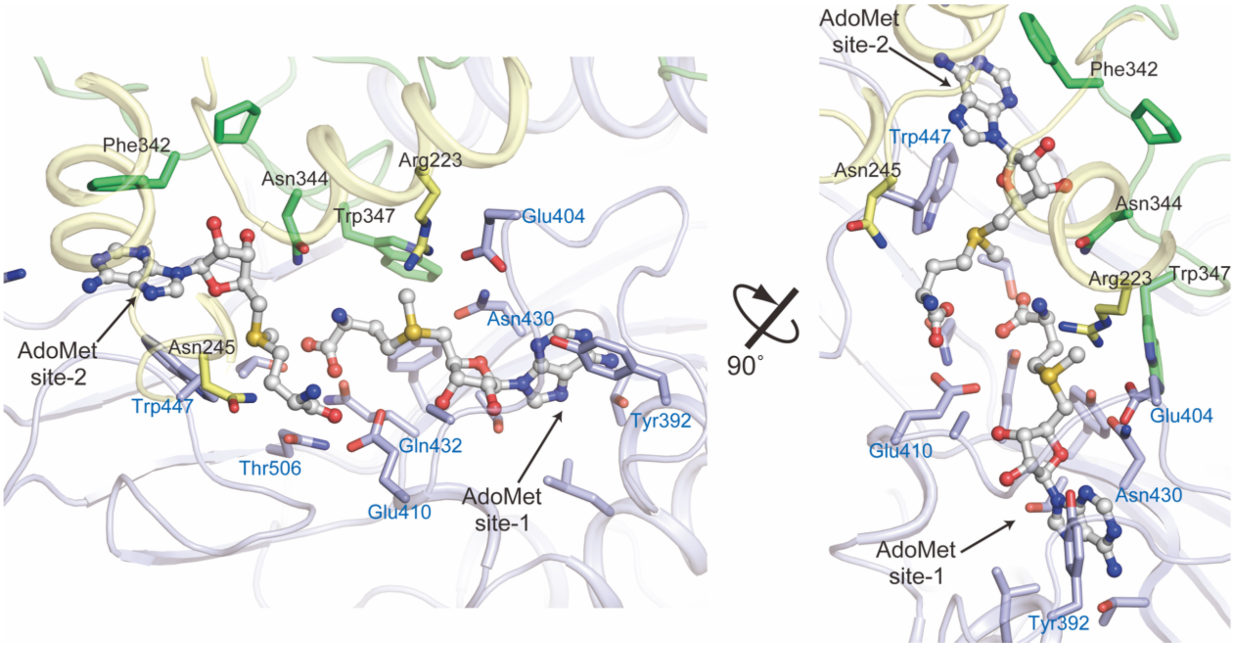


**Supplementary Figure 9. AdoMet binding sites in *c*MTHFR, T-State.** The allosteric inhibitor of *c*MTHFR, AdoMet, is bound in two sites, in the regulatory domain. AdoMet site-1 is the same site AdoHcy occupies in the R-state. AdoMet site-2, the cryptic secondary site, is only unveiled in the T-state, and Phe342 of the velcro-wedge region of the linker and Trp447 of the regulatory domain provide π-stacking interactions that serve to hold the adenine moiety in place.

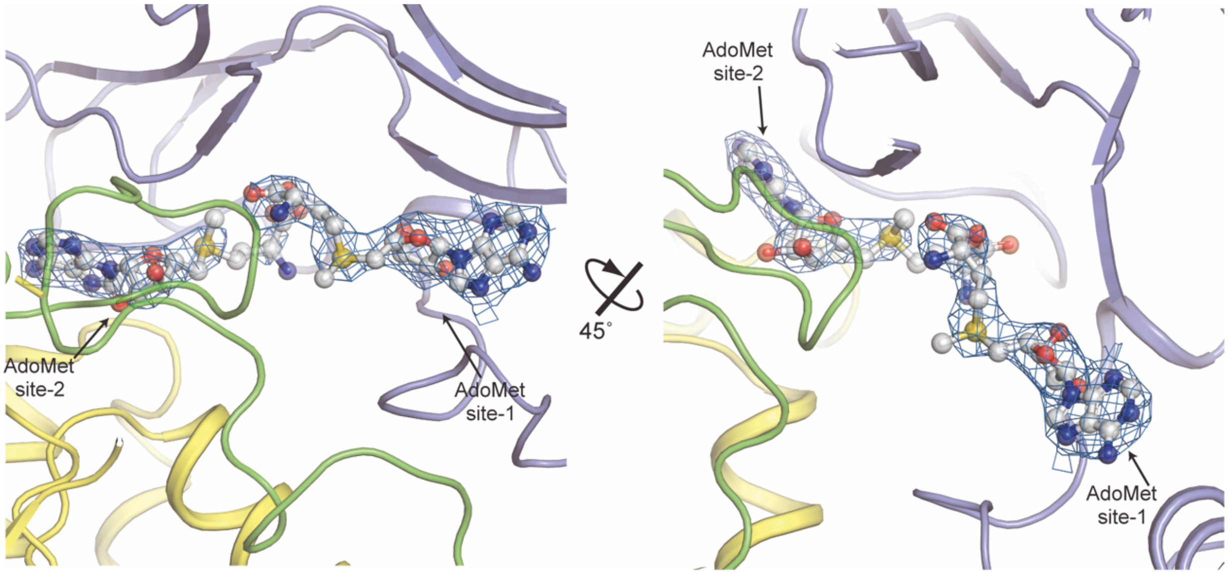


**Supplementary Figure 10. Electron density around AdoMet allosteric inhibitors sites in *c*MTHFR, T-State.** *c*MTHFR, T-State (catalytic domain in yellow and reactivation domain in slate) and the two AdoMet molecules (gray). Their corresponding electron density (2Fo-Fc) contoured at 1.5 σ are shown in blue. The allosteric inhibitor of *c*MTHFR, AdoMet, is bound in two sites in the regulatory domain. AdoMet site-1 is the same site AdoHcy occupies in the R-state. AdoMet site-2, the cryptic secondary site, is only unveiled in the T-state.

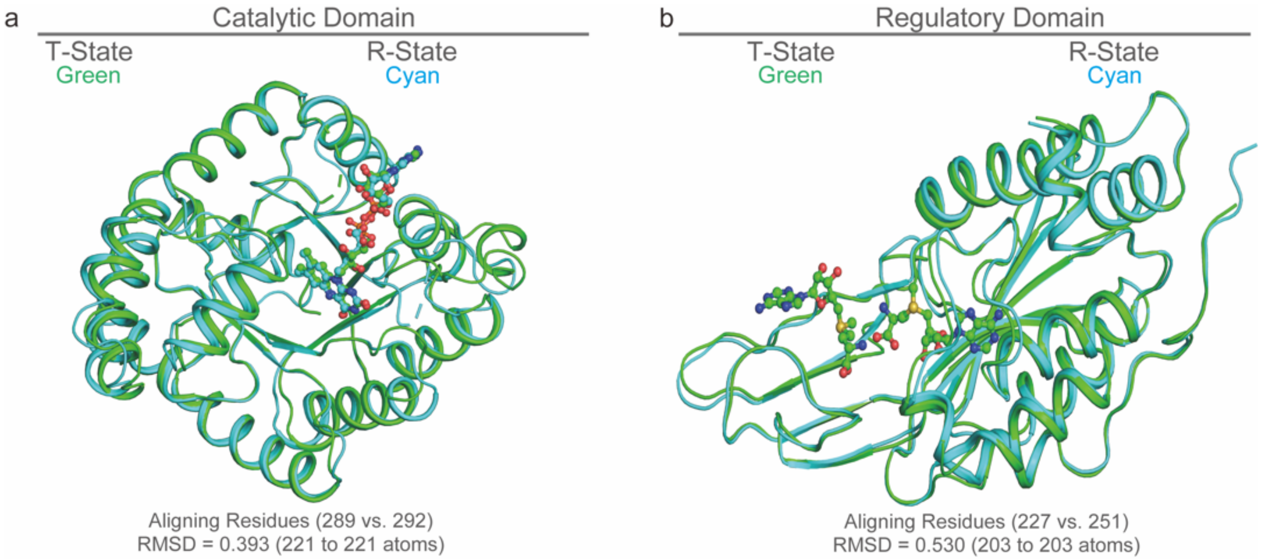


**Supplementary Figure 11. Structural alignment of the catalytic and regulatory domains in the R- and T-states of *c*MTHFR**. **a** The ribbon diagram was used to show the structural alignment of the catalytic domains in *c*MTHFR within the R- and T-states. Cyan and green colors indicate the R and T states, respectively. The FAD cofactors in both structures are shown in stick mode with CMYK colors. **b** The ribbon diagram is used to show the structural alignment of the regulatory domains in *c*MTHFR within the R- and T-states. The color scheme uses cyan for the R-state and green for the T-state. AdoMet ligands bound exclusively in the T-state of *c*MTHFR are shown in stick mode using CMYK colors.

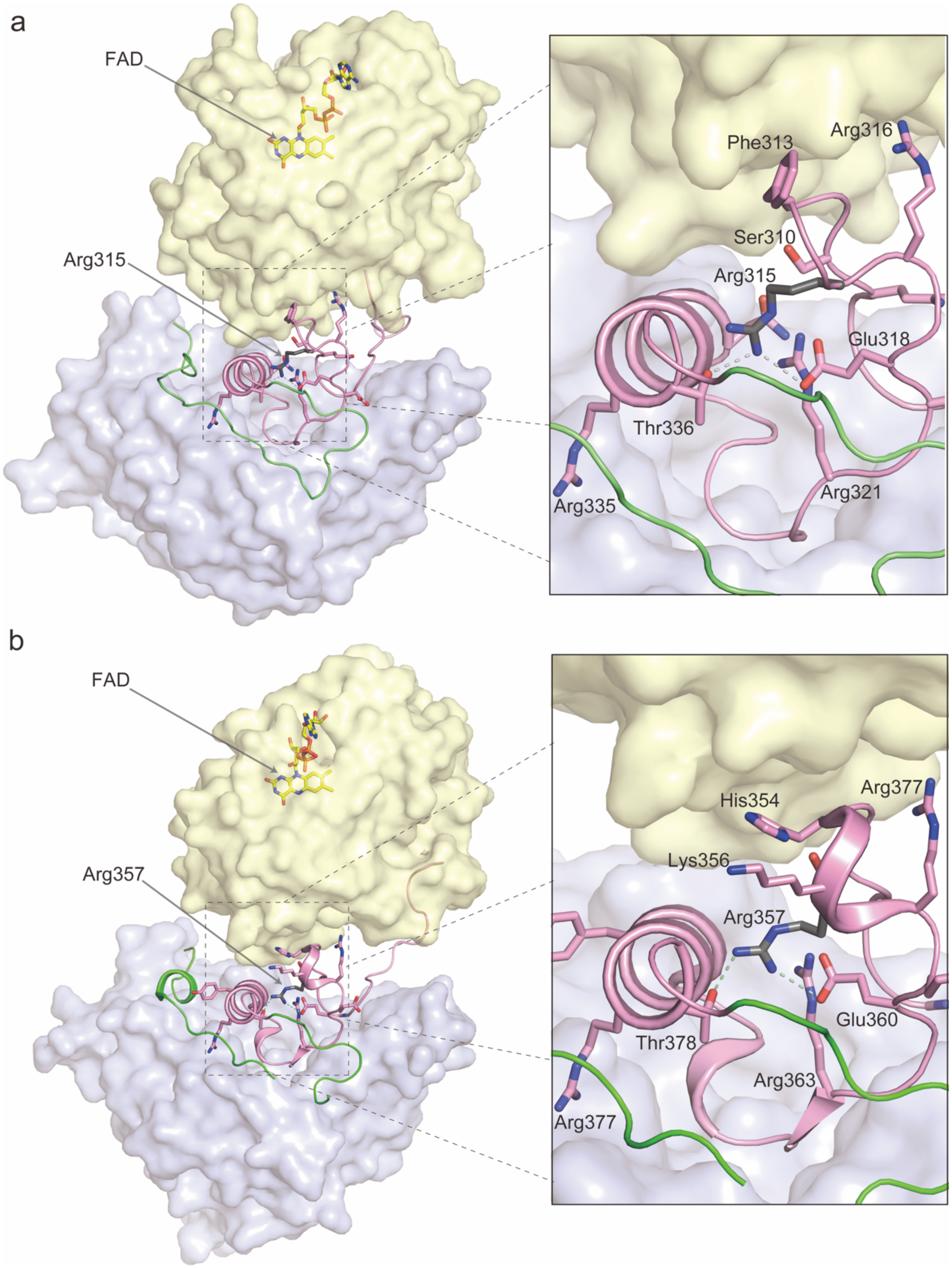


**Supplementary Figure 12. Structural comparison of the role of the linker in interdomain interactions in the R-state of *c*MTHFR and *h*MTHFR**. Structures of *c*MTHFR in the R-state (**a**) and *h*MTHFR in the R-state (**b**) are shown. The catalytic domain and regulatory domain are colored yellow and blue, respectively. The FAD cofactors in both structures are shown in stick mode with CMYK colors. The insets show the position of the Arg residue (315 in *c*MTHFR, 357 in *h*MTHFR) and the retractable-hinge region of the linker (pink), along with positive and polar side chains near it. Loss of this positively charged Arg residue by a Cys mutation (R to C) yields T-state locked MTHFR.

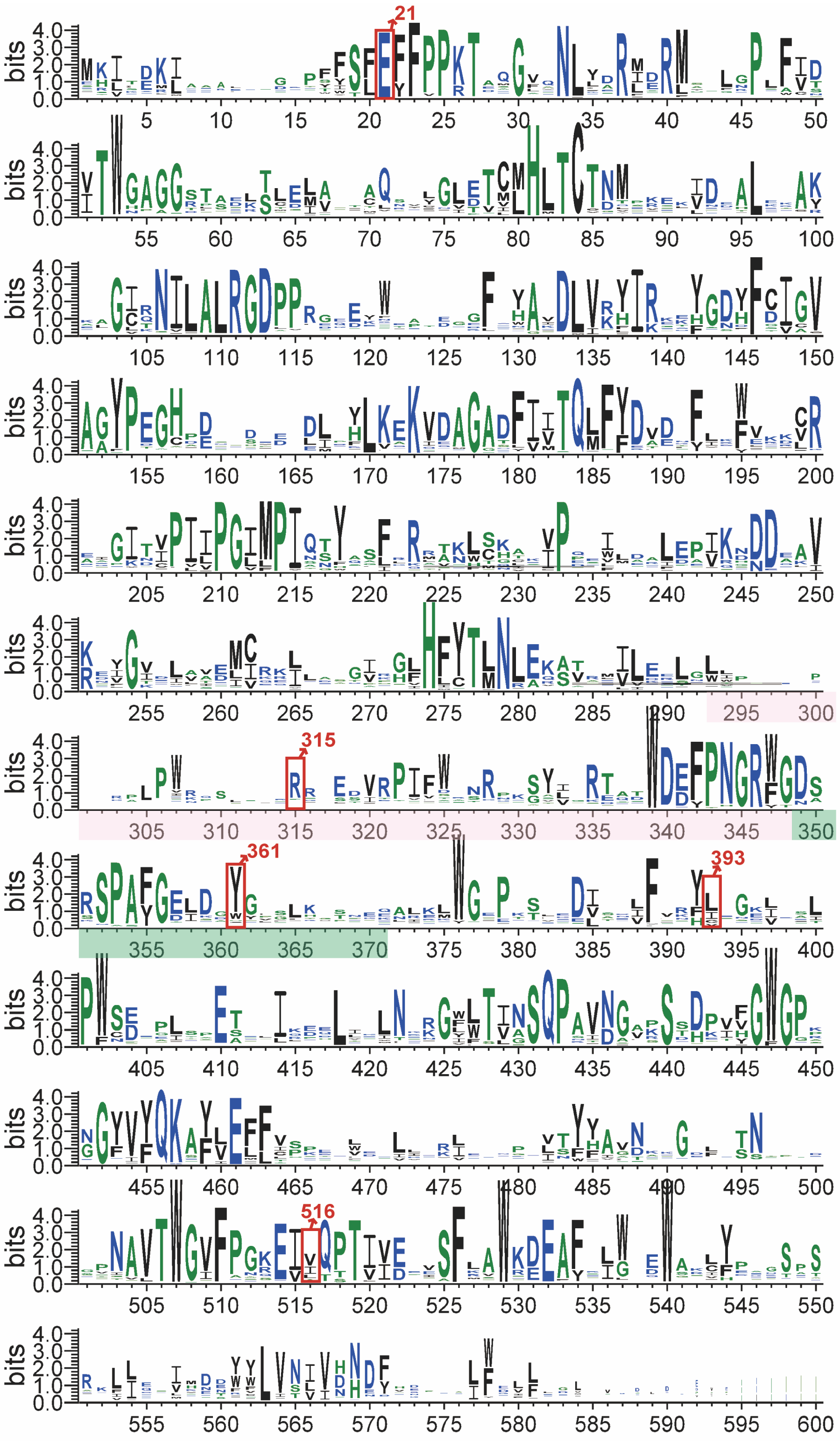


**Supplementary Figure 13. Weblogo representation of a multiple sequence alignment of eukaryotic MTHFRs (n=589).** *c*MTHFR (Uniprot ID G0S5U9) was used as a query sequence. The linker region is colored using the same scheme as in Supplementary Fig. 12. Residue numbering corresponds to that of the fungal enzyme. Residues of importance are indicated by arrows (E21, R315, Y361, L393, and V516). The plot was generated using DeepMSA2[13] and Weblogo3[14].

**Supplementary Table 1. X-Ray Data Collection and Refinement Statistics**

| | $c$MTHFR$^{E21Q, L393M, V516F}$ (R-State) | $c$MTHFR$^{R315A}$ (T-State) |
|---|---|---|
| **Data collection** | | |
| Beamline | APS, LS-CAT 21-IDD | APS, LS-CAT 21-IDD |
| Wavelength (Å) | 1.033 | 1.033 |
| Temperature (K) | 100 | 100 |
| Resolution (Å) | 52.04-3.49 (3.62-3.49)* | 75.09-2.83 (2.89-2.83)* |
| Space group | $P2_12_12_1$ | $P22_12_1$ |
| Cell dimensions | | |
| *a*, *b*, *c* (Å) | 117.97, 151.38, 188.05 | 130.66, 149.95, 171.06 |
| α, β, γ (°) | 90, 90, 90 | 90, 90, 90 |
| Observed reflections | 299,056 (32,269) | 555,396 (32,581) |
| Unique reflections | 43,373 (4,481) | 80,491 (4,556) |
| $R_{meas}$ (%) | 16.4 (218.5) | 14.9 (138.3) |
| $R_{merge}$ (%) | 14.3 (191.1) | 12.6 (117.1) |
| <I/σ> | 8.3 (1.2) | 10.1 (1.9) |
| CC(1/2) | 0.996 (0.562) | 0.987 (0.672) |
| Multiplicity | 6.9 (7.2) | 6.9 (7.2) |
| Completeness (%) | 99.6 (99.9) | 99.8 (100.0) |
| Wilson *B*-factor (Å$^2$) | 50.0 | 52.00 |
| **Refinement** | | |
| Resolution (Å) | 52.04 – 3.49 | 75.09 - 2.83 |
| No. reflections | 41,089 (2,181)‡ | 76,330 (4,071)‡ |
| $R_{work}/R_{free}$ (%) | 26.1/27.2 | 19.3/22.2 |
| No. of non-H atoms | | |
| Protein | 19,081 | 17,836 |
| Water | 4 | 336 |
| Ligand | 212 | 501 |
| B-factors (Å$^2$) | | |
| Protein | 50.0 | 51.3 |
| Water | 80.68 | 61.28 |
| Ligand | 80.68 | 80.98 |
| R.m.s. deviations | | |
| Bond lengths (Å) | 0.0092 | 0.0106 |
| Bond angles (°) | 1.17 | 1.37 |
| Ramachandran Plot | | |
| Favored/allowed/outliers | 97.5/2.4/0.1 | 96.9/2.7/0.4 |
| MolProbity Score | 1.00 (100$^{th}$ percentile) | 1.59 (100$^{th}$ percentile) |
| PDB | 8UY1 | 8UY2 |

* Highest-resolution shell is shown in parentheses.

‡ Number of reflections used for cross-validation

**Supplementary Table 2. Bacterial and insect strains, plasmids, and synthetic oligonucleotides used in this study**

***Strains***

| Cell line | Relevant characteristics | Sources |
|---|---|---|
| *E. coli cells* | | |
| *XL1-Blue* | Routine cloning strain, tetracycline resistance | Stratagene |
| BL21star(DE3) | Widely used T7 expression system, no antibiotic resistance | Invitrogen |
| *Insect cells* | | |
| Sf9 (*Spodoptera frugiperda*) | Host cell for baculovirus-insect cell expression system | Invitrogen |

***Plasmids***

| Names | Relevant characteristics | Ref. or sources |
|---|---|---|
| *Human MTHFR clones* | | |
| pFBHT(hMTHFR$^{wt}$) | wild-type hMTHFR cDNA in pFastBac HT donor vector, encoding an N-terminal His-tag with TEV cleavage site, resistant to ampicillin and gentamicin | Yamada *et al.* 2001[11] |
| pFBHT(hMTHFR$^{A222V}$) | Ala222Val mutant | Yamada *et al.* 2001[11] |
| pFBHT(hMTHFR$^{R357C}$) | Arg357Cys mutant | This Work |
| *Fungal MTHFR clones* | | |
| pMA(cMTHFR$^{wt}$) | synthetic wild-type cMTHFR cDNA in pMA vector, containing the *C. thermophilum* MTHFR cDNA, resistant to ampicillin | GeneArt/Invitrogen (This Work) |
| pMCSG7(cMTHFR$^{wt}$) | wild-type cMTHFR in pMCSG7 vector encoding an N-terminal His-tag with TEV cleavage site, resistant to ampicillin | This Work |
| pMCSG7(cMTHFR$^{R315C}$) | Arg315Cys mutant | This Work |
| pMCSG7(cMTHFR$^{R315A}$) | Arg315Ala mutant | This Work |
| pMCSG7(cMTHFR$^{E21Q, L393M, V516F}$) | Glu21Glu, Leu393Met, and Val516Phe triple-mutant | This Work |

***Oligonucleotides***

| Names | Sequences | Descriptions |
|---|---|---|
| hMTHFR_R357C-f | 5' - CAGTGCACACCCCAAGTGCCGAGAGGAAG - 3' | Site-directed mutagenesis |
| hMTHFR_R357C-r | 5' - CTTCCTCTCGGCACTTGGGGTGTGCACTG - 3' | Site-directed mutagenesis |
| cMTHFR_LIC-f | 5' - TACTTCCAATCCAATGCTATGCATATCCGAGACATGC - 3' | LIC, cMTHFR$^{wt}$ |
| cMTHFR_LIC-r | 5' - TTATCCACTTCCAATGTTAAACTGAGGTCTCCGAAGC - 3' | LIC, cMTHFR$^{wt}$ |
| cMTHFR_E21Q-f | 5' - GCCGTCCTTCTCGTTTCAATACTTCCCGCCCAAGAC - 3' | Site-directed mutagenesis |
| cMTHFR_E21Q-r | 5' - GTCTTGGGCGGGAAGTATTGAAACGAGAAGGACGGC - 3' | Site-directed mutagenesis |
| cMTHFR_R315A-f | 5' - GTCTCTGGGCTTCGGTGCTCGCGGGGAGGATGTCC - 3' | Site-directed mutagenesis |
| cMTHFR_R315A-r | 5' - GGACATCCTCCCCGCGAGCACCGAAGCCCAGAGAC - 3' | Site-directed mutagenesis |
| cMTHFR_R315C-f | 5' - GTCTCTGGGCTTCGGTTGTCGCGGGGAGGATGTCC - 3' | Site-directed mutagenesis |
| cMTHFR_R315C-r | 5' - GGACATCCTCCCCGCGACAACCGAAGCCCAGAGAC - 3' | Site-directed mutagenesis |
| cMTHFR_L393M-f | 5' - CCTCTTCATCCGGTACATGAGAAAGGAAATTGACTAC - 3' | Site-directed mutagenesis |
| cMTHFR_L393M-r | 5' - GTAGTCAATTTCCTTTCTCATGTACCGGATGAAGAGG - 3' | Site-directed mutagenesis |
| cMTHFR_V516F-f | 5' - CCCCGGAAAGGAGATCTTCCAGCCTACCATTGTTGAG - 3' | Site-directed mutagenesis |
| cMTHFR_V516F-r | 5' - CTCAACAATGGTAGGCTGGAAGATCTCCTTTCCGGGG - 3' | Site-directed mutagenesis |

## Supplementary Discussion

### 1. Identification of phosphorylation sites of recombinant wild-type *h*MTHFR

Recombinant *h*MTHFR expressed in insect (Sf9) cells is highly phosphorylated; the phosphoryl groups can be removed by phosphatase treatment[1,2]. In the present study, we found 11 phosphorylation sites in *h*MTHFR. Although 16 phosphorylation sites were previously reported[2], including non-surface exposed sites such as Tyr90 in the catalytic domain, all amino acid residues identified by our analysis were exposed to solvent based on the *h*MTHFR structure (PDB, 6FCX)[2]. A summary of phosphorylation sites in recombinant wild-type *h*MTHFR in previously reported and our analyses is shown (Supplementary Fig. 1). There are eight overlapping residues in the identified phosphorylation sites. Of particular significance are the seven residues within this overlap that populate the N-terminal Ser/Thr rich region, namely, Ser21, Ser23, Ser25, Ser26, Ser29, Ser30, and Thr34. The latter is thought to be the priming position for post-translational modification of *h*MTHFR by Pro-directed kinase(s). An additional phosphorylation site, Ser394, is located within the linker region. Kinases, including CDK1/cyclin B[3], polo-like kinase 1[4], DYRK1A/2[5], and GSK3A/B[5], have been proposed to participate in the post-translational modification of *h*MTHFR.

Our LC-MS analysis revealed 11 phosphorylation sites in recombinant *h*MTHFR. More than two trypsin-digested phosphopeptide fragments were used to confirm these modifications required the use of the higher-energy collisional dissociation (HCD)-induced fragmentation mass spectra, facilitated phosphopeptide identification. Typical MS/MS data are shown (Supplementary Fig. 2).
AdoMet acts a more conspicuous inhibitory influence on the MTHFR activity of fully modified *h*MTHFR in contrast to phosphatase-treated *h*MTHFR at reduced concentrations. Furthermore, *h*MTHFR$^{T34A}$ also manifests in reduced responsiveness to AdoMet, possibly due to the Thr34 to Ala substitution impeding sequential post-translational modifications. This observation posits an association between the post-translational modification of *h*MTHFR and the allosteric regulation and transition of the enzyme. Moreover, in light of the attenuated sensitivity to AdoMet observed in the N-terminal truncated *h*MTHFR[2], it becomes apparent that the heavily phosphorylated N-terminal region assumes a pivotal role in the allosteric transition of *h*MTHFR to the T-state. The role of the linker remains unclear though it is thought to interact with the phosphorylated amino acid side chains of the N-terminal, albeit in a differential mode depending on the conformation (R vs. T-state).

## 2. *h*MTHFR Arg357Cys patient mutation & other patient mutations

Over 100 mutations have been reported in the human MTHFR gene[6]. The Arg357Cys mutation in the MTHFR deficient patients results from the rare 1081C>T gene mutation[6,7,8]. The numbering of nucleotide and amino acid residues follows the nomenclature of the previous report[7]. The 1081C>T gene mutation is a rare mutation; it has been found in nine alleles in two families[6]. Patients with 1081C>T have low MTHFR activities, 5~27% of controls; the activity range of those with MTHFR deficiency is 0%-20% to controls. Thus, most of them experience severe MTHFR deficiency[9,10].

The recombinant histidine-tagged *h*MTHFR Arg357Cys mutant was produced by a previously reported method employing the baculovirus-insect cell expression system[11]. The Arg357Cys mutant is primarily found in the soluble fraction of the protein (Supplementary Fig. 3a). The Arg357Cys mutant showed a slower mobility on SDS-PAGE compared to the Thr34Ala mutant, which had no post-translational modifications[11]. Rather, it migrates with similar mobility as the wild-type enzyme. Therefore, it is likely that the Arg357Cys mutant undergoes a post-translational modification, such as phosphorylation[11].

However, the cell extract expressing the *h*MTHFR Arg357Cys mutant showed only ~8% NADPH-menadione oxidoreductase activity compared to that expressing wild-type *h*MTHFR, and it was only slightly higher than the endogenous NADPH oxidase activity in the Sf9 cell. It is generally known that the NADPH-menadione oxidoreductase assay is not suitable for estimating recombinant MTHFR enzyme activity in cell lysate. However, our expression system allowed us to roughly estimate the recombinant MTHFR enzyme activity in the cell lysate due to the high level of the protein production. Routinely, NADPH-menadione oxidoreductase activity in uninfected Sf9 cells, a negative control for non-MTHFR-related activity, is less than ≈5% of that in baculovirus-infected Sf9 cells expressing wild-type *h*MTHFR. The high-level expression of *h*MTHFR facilitated the acquisition of purified recombinant mutants. Despite the use of the purified enzyme, the mutant exhibited an extremely low turnover number (Supplementary Fig. 3b). Due to this low activity, the determination of NADPH affinity and AdoMet inhibition could not be determined.

The purified Arg357Cys mutant, which had a typical flavin spectrum, was subjected to FAD release measurement from *h*MTHFR, according to a previously reported method with minor modifications[11]. Briefly, a spectrofluorophotometer RF-5300PC (Shimadzu, Kyoto, Japan) equipped with a cell temperature controller monitored the released FAD from *h*MTHFR. The excitation and emission wavelengths were set at 390 nm and 525 nm, respectively. Concentrated MTHFR was diluted directly into pre-warmed 50 mM KPB at pH 7.2 (3 mL) at 46 ˚C, and the fluorescence intensity of released FAD was monitored for 10 minutes. FAD release from *h*MTHFR$^{R357C}$ was measured across varied concentrations (50~400 nM). At 100 nM, the rate constant of FAD release from the Arg357Cys mutant was 0.030 sec$^{-1}$, which is comparable to that of the wild-type enzyme at 0.031 sec$^{-1}$ (Supplementary Fig. 3c, inset). The Ala222Val common variant, a well-known thermolabile protein associated with mild hyperhomocysteinemia, was used as a model for the faster FAD release mutant[11]. Consequently, it was evident that *h*MTHFR$^{R357C}$ binds the FAD cofactor as tightly as the wild-type enzyme, while impairing the catalytic function. However, the mutation, located in the linker region away from the active site, posed a challenge in attributing the involvement of the Arg357 residue to one of the catalytic residues.

FAD release from *h*MTHFR is affected by AdoMet, as previously reported[11]. The initial rate of FAD release is decreased in the presence of AdoMet; presumably, in the inhibited state, the T-state, *h*MTHFR resists releasing FAD. This gives rise to the idea that the Arg357Cys mutation in the linker region alters the protein conformation to the T-state to inhibit the enzyme activity, rather than affecting the catalytic function as one of the catalytic residues in the active site. The protein conformation of *h*MTHFR$^{R357C}$ is related to the T-state even in the absence of AdoMet, which could explain the properties of the mutant. Taken together, we postulated that the *h*MTHFR$^{R357C}$ patient mutant represents a "T-state locked" enzyme.

### 3. Limited proteolysis of *c*MTHFR$^{wt}$ and *c*MTHFR$^{R315A}$ in the presence or absence of AdoMet

Conformational changes of *c*MTHFR$^{wt}$, in the presence or absence of AdoMet, were visualized by limited proteolysis followed by SDS-PAGE (Supplementary Fig. 6a)[12]. Our analysis suggests that the retractable region in the linker (pink, Supplementary Fig. 6b) should be exposed to the solvent when AdoMet binds to *c*MTHFR$^{wt}$. This configuration allows trypsin to digest the linker (Supplementary Fig. 6c). In contrast, in the R-state, MTHFR securely folds the retractable region between the catalytic and regulatory domains, preventing trypsin cleavage. In the absence of AdoMet, a fraction of *c*MTHFR$^{wt}$ evades tryptic digestion due to the conformational equilibrium between the R- and T-state. In the presence of AdoMet, *c*MTHFR$^{wt}$ exhibits increased susceptibility to a lower amount of trypsin, indicating that AdoMet induces a conformational shift to the T-state where trypsin successfully digests the retractable region exposed to the solvent.

Conformational changes of *c*MTHFR$^{R315A}$, in the presence or absence of AdoMet, were visualized by limited proteolysis followed by SDS-PAGE (Supplementary Fig. 7a)[12]. Our analysis suggests that the retractable region in the linker (pink, Supplementary Fig. 7b) should be exposed to the solvent when AdoMet binds to *c*MTHFR (i.e. is found in the T-state). This configuration allows trypsin to digest the linker (Supplementary Fig. 7c). In the absence of AdoMet, a fraction of *c*MTHFR$^{R315A}$ evades tryptic digestion due to the conformational equilibrium between the R- and T-state. In the presence of AdoMet, *c*MTHFR$^{R315A}$ does not exhibit any significant difference, indicating that *c*MTHFR$^{R315A}$ is already conformational shifted to the T-state, where trypsin successfully digests the retractable region exposed to the solvent. *c*MTHFR$^{R315A}$ is locked in the T-state, demonstrating no change in conformation in the presence or absence of AdoMet.

**4. Structural rigidity of *c*MTHFR domains in R and T-states**

Notwithstanding the profound distinctions in protein conformation observed in the R- and T-states of *c*MTHFR structures, the catalytic and regulatory domains exhibited a propensity for rigidity rather than flexibility. The comparative analysis of *c*MTHFR domains in the R-state and the T-state (Supplementary Fig. 11). The topological configuration of the MTHFR catalytic domain remains conserved across different organisms, ranging from bacterial enzymes to higher eukaryotic enzymes. This domain has the shape of a $\beta_8\alpha_8$ barrel, constituting the TIM barrel fold and comprises approximately 290 amino acid residues. The catalytic domains of *c*MTHFR were aligned in both the R- and T-states (Supplementary Fig. 11a). The root mean square deviation (RMSD) was calculated to be 0.393, using 221 α-carbon atoms. The structural topology of the regulatory domain presents a unique fold exclusively identified in eukaryotic MTHFR[2], and absent in bacterial MTHFR. The regulatory domains of *c*MTHFR in both R- and T-states were superimposed (Supplementary Fig. 11b), using 226 residues for the alignment. The RMSD values, based on 203 α-carbon atoms, are impressively low at 0.530. These results underscore the inherent rigidity that characterizes the domain architecture in both the R- and T-states of *c*MTHFR. Consequently, it is suggested that the flexibility inherent in the linker region facilitates the substantial conformational transition observed between the R- and T-state structures.

## References


1. Yamada, K., Strahler, J. R., Andrews, P. C. & Matthews, R. G. Regulation of human methylenetetrahydrofolate reductase by phosphorylation. *Proc. Natl. Acad. Sci.* **102**, 10454–10459 (2005).
2. Froese, D. S. *et al.* Structural basis for the regulation of human 5,10-methylenetetrahydrofolate reductase by phosphorylation and S-adenosylmethionine inhibition. *Nat. Commun.* **9**, 2261 (2018).
3. Zhu, B. *et al.* MTHFR promotes heterochromatin maintenance. *Biochem. Biophys. Res. Commun.* **447**, 702–706 (2014).
4. Li, X. *et al.* Polo-like kinase 1 (PLK1)-dependent phosphorylation of methylenetetrahydrofolate reductase (MTHFR) regulates replication via histone methylation. *Cell Cycle* **16**, 1933–1942 (2017).
5. Zheng, Y. *et al.* Regulation of folate and methionine metabolism by multisite phosphorylation of human methylenetetrahydrofolate reductase. *Sci. Rep.* **9**, 4190 (2019).
6. Froese, D. S. *et al.* Mutation Update and Review of Severe Methylenetetrahydrofolate Reductase Deficiency. *Hum. Mutat.* **37**, 427–438 (2016).
7. Goyette, P., Frosst, P., Rosenblatt, D. S. & Rozen, R. Seven novel mutations in the methylenetetrahydrofolate reductase gene and genotype/phenotype correlations in severe methylenetetrahydrofolate reductase deficiency. *Am. J. Hum. Genet.* **56**, 1052–1059 (1995).
8. Tonetti, C., Burtscher, A., Bories, D., Tulliez, M. & Zittoun, J. Methylenetetrahydrofolate reductase deficiency in four siblings: A clinical, biochemical, and molecular study of the family. *Am. J. Med. Genet.* **91**, 363–367 (2000).
9. Rosenblatt, D. S. Inherited Disorders of Folate and Cobalamin. in *Homocysteine Metabolism: From Basic Science to Clinical Medicine* (eds. Graham, I., Refsum, H., Rosenberg, I. H., Ueland, P. M. & Shuman, J. M.) 61–68 (Springer US, Boston, MA, 1997). doi:10.1007/978-1-4615-5771-5_9.
10. Goyette, P., Christensen, B., Rosenblatt, D. S. & Rozen, R. Severe and mild mutations in cis for the methylenetetrahydrofolate reductase (MTHFR) gene, and description of five novel mutations in MTHFR. *Am. J. Hum. Genet.* **59**, 1268–1275 (1996).
11. Yamada, K., Chen, Z., Rozen, R. & Matthews, R. G. Effects of common polymorphisms on the properties of recombinant human methylenetetrahydrofolate reductase. *Proc. Natl. Acad. Sci.* **98**, 14853–14858 (2001).
12. Matthews, R. G., Vanoni, M. A., Hainfeld, J. F. & Wall, J. Methylenetetrahydrofolate reductase. Evidence for spatially distinct subunit domains obtained by scanning transmission electron microscopy and limited proteolysis. *J. Biol. Chem.* **259**, 11647–11650 (1984).
13. Zheng, W. *et al.* Improving deep learning protein monomer and complex structure prediction using DeepMSA2 with huge metagenomics data. *Nat. Methods* **21**, 279–289 (2024).
14. Crooks, G. E., Hon, G., Chandonia, J.-M. & Brenner, S. E. WebLogo: A Sequence Logo Generator. *Genome Res.* **14**, 1188–1190 (2004).
