# Supplementary material for: Structural basis of S-adenosylmethionine-dependent allosteric transition from active to inactive states in methylenetetrahydrofolate reductase
Source: Nat Commun. 2024 Jun 17;15:5167. doi: 10.1038/s41467-024-49327-5 (PMC11183114; doi:10.1038/s41467-024-49327-5)
Supplement: Supplementary file 3 — Reporting Summary [file 41467_2024_49327_MOESM3_ESM.pdf]

nature portfolio

Corresponding author(s): Markos Koutmos

Last updated by author(s): 05/23/2024

Reporting Summary

Nature Portfolio wishes to improve the reproducibility of the work that we publish. This form provides structure for consistency and transparency in reporting. For further information on Nature Portfolio policies, see our Editorial Policies and the Editorial Policy Checklist.

Statistics

For all statistical analyses, confirm that the following items are present in the figure legend, table legend, main text, or Methods section.

n/a	Confirmed	
☐	☒	The exact sample size (*n*) for each experimental group/condition, given as a discrete number and unit of measurement
☐	☒	A statement on whether measurements were taken from distinct samples or whether the same sample was measured repeatedly
☒	☐	The statistical test(s) used AND whether they are one- or two-sided<br>*Only common tests should be described solely by name; describe more complex techniques in the Methods section.*
☒	☐	A description of all covariates tested
☒	☐	A description of any assumptions or corrections, such as tests of normality and adjustment for multiple comparisons
☒	☐	A full description of the statistical parameters including central tendency (e.g. means) or other basic estimates (e.g. regression coefficient) AND variation (e.g. standard deviation) or associated estimates of uncertainty (e.g. confidence intervals)
☒	☐	For null hypothesis testing, the test statistic (e.g. *F*, *t*, *r*) with confidence intervals, effect sizes, degrees of freedom and *P* value noted<br>*Give P values as exact values whenever suitable.*
☒	☐	For Bayesian analysis, information on the choice of priors and Markov chain Monte Carlo settings
☒	☐	For hierarchical and complex designs, identification of the appropriate level for tests and full reporting of outcomes
☒	☐	Estimates of effect sizes (e.g. Cohen's *d*, Pearson's *r*), indicating how they were calculated

Our web collection on statistics for biologists contains articles on many of the points above.

Software and code

Policy information about availability of computer code

Data collection	UV-Vis data were collected on an Agilent Cary 100 Bio spectrophotometer with Agilent Cary WinUV software.<br>Fluorescence data were collected on a Shimadzu RF-5300PC spectrofluorophotometer with Shimadzu LabSolutions RF software.<br>LC-MS/MS data were collected on a Thermo Scientific Orbitrap Fusion Lumos Tribrid MS coupled with the UltiMate 3000 RSLCnano liquid chromatography system with Thermo Scientific Proteome Discoverer software.<br>X-ray crystallography data collection was performed using the LS-CAT beamline 21-ID-D at the Advanced Photon Source, Argonne National Laboratory (Argonne, IL). The beam line/data collection were controlled using their standard in-house user interface.
Data analysis	CCP4 8.0.016, AIMLESS 0.5.21, PHASER 2.8.3, xia2/DIALS 0.3.8.0, BUSTER 2.10.4, Phenix 1.20.1-4487, Coot 0.9.8.1, PDB-REDO, MolProbity 2.5, PyMol 2.5.4, Graph Pad Prism 10.2.3, Kaleidagraph 4.1.4, Illustrator 28.0, ProteomeDiscoverer 2.2.0.388

For manuscripts utilizing custom algorithms or software that are central to the research but not yet described in published literature, software must be made available to editors and reviewers. We strongly encourage code deposition in a community repository (e.g. GitHub). See the Nature Portfolio guidelines for submitting code & software for further information.

Data

Policy information about availability of data

All manuscripts must include a data availability statement. This statement should provide the following information, where applicable:

- Accession codes, unique identifiers, or web links for publicly available datasets
- A description of any restrictions on data availability
- For clinical datasets or third party data, please ensure that the statement adheres to our policy

The structure coordinates and structure factors reported in this study have been deposited in the Protein Data Bank under accession codes 8UY1 [http://doi.org/10.2210/pdb8uy1/pdb] (cMTHFR E21Q, L393M, V516F) and 8UY2 [http://doi.org/10.2210/pdb8uy2/pdb] (cMTHFR R315A) . PDB codes of previously published structures used in this study are 6FCX [http://doi.org/10.2210/pdb6fcx/pdb]. The mass spectrometry phosphorylation data set and raw files have been deposited to the MassIVE repository under accession number MSV000094828 [https://doi.org/doi:10.25345/C5DZ03C7F]. All other data are available from the corresponding authors upon request. Source data are provided in this paper.

All other relevant data pertaining to this study are available in the Source data, provided as a Source Data File, and in the Supplementary Information.

Research involving human participants, their data, or biological material

Policy information about studies with human participants or human data. See also policy information about sex, gender (identity/presentation), and sexual orientation and race, ethnicity and racism.

Reporting on sex and gender	N/A
Reporting on race, ethnicity, or other socially relevant groupings	N/A
Population characteristics	N/A
Recruitment	N/A
Ethics oversight	N/A

Note that full information on the approval of the study protocol must also be provided in the manuscript.

Field-specific reporting

Please select the one below that is the best fit for your research. If you are not sure, read the appropriate sections before making your selection.

☒ Life sciences ☐ Behavioural & social sciences ☐ Ecological, evolutionary & environmental sciences

For a reference copy of the document with all sections, see nature.com/documents/nr-reporting-summary-flat.pdf

Life sciences study design

All studies must disclose on these points even when the disclosure is negative.

Sample size	Sample size was not predetermined. Unless otherwise stated, N=2 (two independent experiments) were used for enzyme/functional assays.
Data exclusions	No data points were excluded from analyses.
Replication	At least three independent experiments were conducted for the functional assays. All attempts at replication were successful. The representative results are displayed in Figure 2a-d, for Supplementary Figure 3b and c, and for Supplementary Figure 6a and 7a.
Randomization	No randomization was performed as data was collected without the need for experimental group allocation.
Blinding	No blinding was performed as no experimental group allocation was necessary.

Reporting for specific materials, systems and methods

We require information from authors about some types of materials, experimental systems and methods used in many studies. Here, indicate whether each material, system or method listed is relevant to your study. If you are not sure if a list item applies to your research, read the appropriate section before selecting a response.

Materials & experimental systems

n/a	Involved in the study
☒	☐ Antibodies
☐	☒ Eukaryotic cell lines
☒	☐ Palaeontology and archaeology
☒	☐ Animals and other organisms
☒	☐ Clinical data
☒	☐ Dual use research of concern
☒	☐ Plants

Methods

n/a	Involved in the study
☒	☐ ChIP-seq
☒	☐ Flow cytometry
☒	☐ MRI-based neuroimaging

Eukaryotic cell lines

Policy information about cell lines and Sex and Gender in Research

Cell line source(s)	Sf9 cells (Expression system: 10359-016, cells: 11496-015)
Authentication	Cell lines were not authenticated as they were purchased less than a year ago
Mycoplasma contamination	Cell lines were not test for mycoplasma contamination as they were purchased less than a year ago
Commonly misidentified lines (See ICLAC register)	None were used in this study

Plants

Seed stocks	N/A
Novel plant genotypes	N/A
Authentication	N/A
